# Xiaoqinglong decoction mitigates nasal inflammation and modulates gut microbiota in allergic rhinitis mice

**DOI:** 10.3389/fmicb.2024.1290985

**Published:** 2024-05-15

**Authors:** Hao-Lan Liu, Hui-Fang Chen, Yun-Dang Wu, Ya-Jie Yan, Xue-Cheng He, Zhong-Zheng Li, Yan Ruan, Gan-Long Wu

**Affiliations:** ^1^School of Medicine, Jishou University, Jishou, China; ^2^Department of Otolaryngology Head and Neck Surgery, The First Affiliated Hospital of Guangzhou University of Chinese Medicine, Guangzhou, China; ^3^Lingnan Institute of Otolaryngology, Guangdong Clinical Research Academy of Chinese Medicine, Guangzhou, China; ^4^Department of Medicine, Guangxi University of Science and Technology, Liuzhou, China; ^5^National-Regional Joint Engineering Research Center for Soil Pollution Control and Remediation in South China, Guangzhou, China; ^6^Guangdong Key Laboratory of Integrated Agro-environmental Pollution Control and Management, Institute of Eco-environmental and Soil Sciences, Guangdong Academy of Sciences, Guangzhou, China; ^7^The First Clinical Medical College of Guangzhou University of Chinese Medicine, Guangzhou, China; ^8^People’s Hospital of Jishou City, Jishou, China

**Keywords:** Xiaoqinglong decoction, allergic rhinitis, type 2 inflammation, gut microbiota, gut-lung axis

## Abstract

**Introduction:**

Allergic rhinitis (AR) is a respiratory immune system disorder characterized by dysregulation of immune responses. Within the context of AR, gut microbiota and its metabolites have been identified as contributors to immune modulation. These microorganisms intricately connect the respiratory and gut immune systems, forming what is commonly referred to as the gut-lung axis. Xiaoqinglong Decoction (XQLD), a traditional Chinese herbal remedy, is widely utilized in traditional Chinese medicine for the clinical treatment of AR. In this study, it is hypothesized that the restoration of symbiotic microbiota balance within the gut-lung axis plays a pivotal role in supporting the superior long-term efficacy of XQLD in AR therapy. Therefore, the primary objective of this research is to investigate the impact of XQLD on the composition and functionality of the gut microbiota in a murine model of AR.

**Methods:**

An ovalbumin-sensitized mouse model to simulate AR was utilized, the improvement of AR symptoms after medication was investigated, and high-throughput sequencing was employed to analyze the gut microbiota composition.

**Results:**

XQLD exhibited substantial therapeutic effects in AR mice, notably characterized by a significant reduction in allergic inflammatory responses, considerable alleviation of nasal symptoms, and the restoration of normal nasal function. Additionally, following XQLD treatment, the disrupted gut microbiota in AR mice displayed a tendency toward restoration, showing significant differences compared to the Western medicine (loratadine) group.

**Discussion:**

This results revealed that XQLD may enhance AR allergic inflammatory responses through the regulation of intestinal microbiota dysbiosis in mice, thus influencing the dynamics of the gut-lung axis. The proposal of this mechanism provides a foundation for future research in this area.

## 1 Introduction

Respiratory tract infections are frequently accompanied by disruptions in the gut microbiota, which subsequently trigger immune-inflammatory responses. The gut microbiota and its metabolic byproducts actively contribute to the host’s immune regulation, particularly within the context of respiratory conditions (Chakradhar, 2017). Researchers have introduced the term gut-lung axis to elucidate the intricate interrelationship between the respiratory and gastrointestinal tracts through microbiota interactions, effectively connecting the immune systems of the lungs and intestines (Borchers et al., 2009; Stricker et al., 2022).

Recent research reveals the intricate role of gut microbiota and its metabolites in modulating allergic rhinitis (AR)-related immune responses. Genetic mutations governing immune recognition, adaptive immunity, and epithelial cell permeability are influenced by the gut microbiota in human and murine studies, disrupting microbiota and impacting immune homeostasis. This disruption contributes significantly to allergic reactions and inflammation (Woo and Alenghat, 2017). The immune system, in turn, plays a multifaceted role in regulating the balance between the host and its microbiota. Therefore, respiratory diseases often coincide with gut microbiota dysbiosis and immune-inflammatory responses (Ma et al., 2022). Investigations show altered gut microbiota diversity in pediatric and adult AR patients (Lin et al., 2020). Disturbances in the gut microbiota reciprocally influence the respiratory tract, with disrupted gut microbiota in mice leading to heightened susceptibility to respiratory allergic reactions. Interventions with probiotics in AR mice have shown promise in mitigating airway hyperreactivity, reducing inflammation, and lowering allergen-specific IgE production (Fiocchi et al., 2022). These findings emphasize the crucial regulatory role of gut microbiota in AR pathophysiology (Ma et al., 2022).

Xiaoqinglong Decoction, a widely used traditional Chinese medicinal formula for treating allergic disorders like AR and asthma, is notable for its remarkable therapeutic effectiveness and low recurrence rates. Multiple clinical trials consistently demonstrate its substantial benefits compared to control medications. In a 6-month treatment, AR patients on XQLD had a 7.54% recurrence rate, much lower than the loratadine-treated control group’s 26.4% (Chen, 2012). A rigorous 8-week trial post-XQLD treatment showed no notable adverse reactions (Kim et al., 2019). Symptomatology and physical indicators consistently improved compared to the control group. Considering gut microbiota research, AR disrupts the microbiota balance in the gut-lung axis, making immune homeostasis more susceptible to perturbation. This vulnerability likely contributes to AR recurrence and limited efficacy of conventional interventions. Understanding XQLD’s impact on gut microbiota during AR treatment has significant implications for its enduring therapeutic effects. Notably, no investigations have explored XQLD’s regulatory impact on gut microbiota in the context of AR to date.

Expanding on this foundation, our study advances a hypothesis: the potential augmentation of long-term therapeutic efficacy in AR treatment with XQLD may be attributed to the restoration of symbiotic microbiota balance within the gut-lung axis. XQLD holds significant promise for modulating and preserving gut microbiota, setting it apart from conventional pharmaceutical interventions. To investigate this hypothesis, we established an AR mouse model and administered XQLD as an intervention. We conducted a comprehensive examination of the therapeutic effects of XQLD on AR mice, with a specific focus on the analysis of gut microbiota alterations following XQLD treatment. These findings shed light on the transformations within the mouse gut microbiota influenced by XQLD, providing valuable insights for its clinical application in the treatment of AR.

## 2 Materials and methods

### 2.1 Mouse preparation

We induced a systemic immune response in mice by intraperitoneal injection of ovalbumin (OVA) combined with aluminum hydroxide solution. Then, AR symptoms were induced by intranasal sensitization with OVA solution. Because AR is mediated by IgE, which can induce Th2 cell differentiation and polarization, resulting in symptoms such as nasal congestion, sneezing, and clear rhinorrhea, we evaluated the success of the mouse AR model after modeling by observing nasal symptoms and measuring serum levels of OVA-specific IgE, as well as the expression levels of Th2-related factors interleukin 4 (IL-4), interleukin 5 (IL-5), and interleukin 13 (IL-13). The detailed information is as follows.

Female C57BL/6 mice, aged 6–8 weeks and weighing 18–22 g, were procured from Changsha Tianqin Biotechnology Co., Ltd. [License: SCXK (Xiang) 2019–0014]. The mice were housed in a specific pathogen-free (SPF) animal facility at the Experimental Animal Center of the First Affiliated Hospital of Guangzhou University of Chinese Medicine [License: SYXK (Yue) 018–0092]. Ethical approval for this study was obtained from the Ethics Committee of the Animal Experimental Center at the First Affiliated Hospital of Guangzhou University of Chinese Medicine under approval number TCMF1-2019048. The mice were randomly allocated into 4 groups: the blank control group, AR model group, Loratadine group, XQLD group, with each group comprising eight mice. The reason we chose loratadine is because loratadine is the most commonly used medication for treating allergic rhinitis. It is a second-generation selective H1 receptor antagonist that can reduce histamine release, decrease peripheral capillary permeability, reduce tissue swelling, and counteract allergic reactions, thereby alleviating symptoms of AR such as nasal congestion, sneezing, and rhinorrhea.

**Figure 1:**
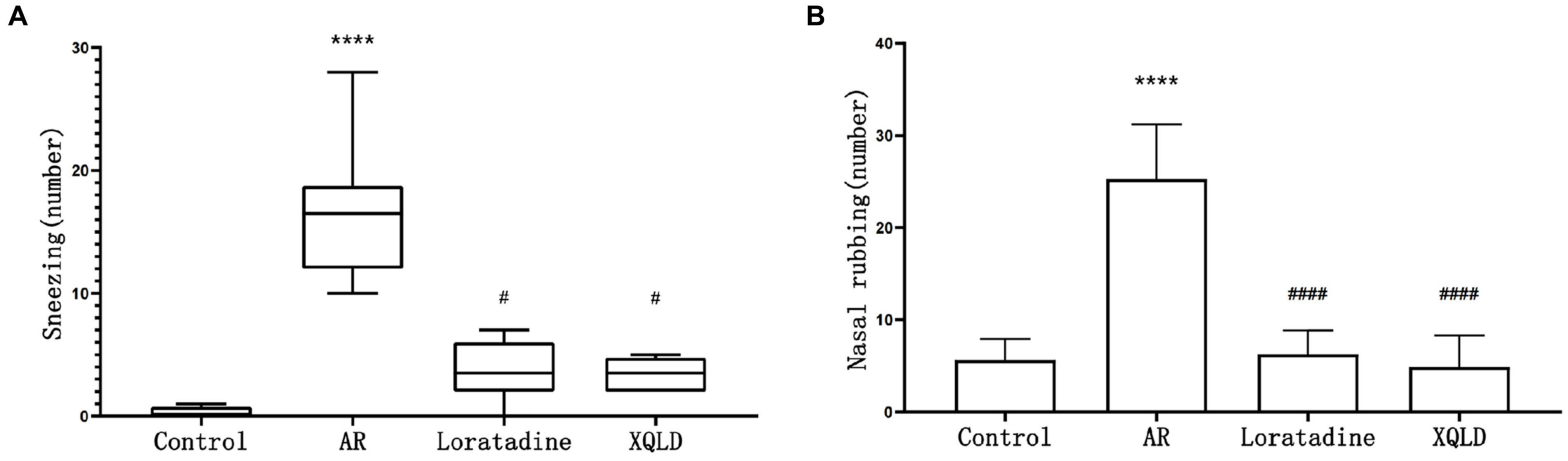
Incidence of nasal allergic symptoms in mice-sneezing **(A)** and nose rubbing **(B)**. The significance analysis of the AR group is obtained by comparing it with the control group to obtain the *p*-value (^*^p < 0.05 and ^****^p < 0.0001). The significance analysis of the Loratadine and XQLD groups is obtained by comparing them with the AR group to obtain the *p*-value (^#^*p* < 0.05 and ^####^*p* < 0.0001). Control refers to the group where no drug treatment is administered after the modeling process.

**Figure 2:**
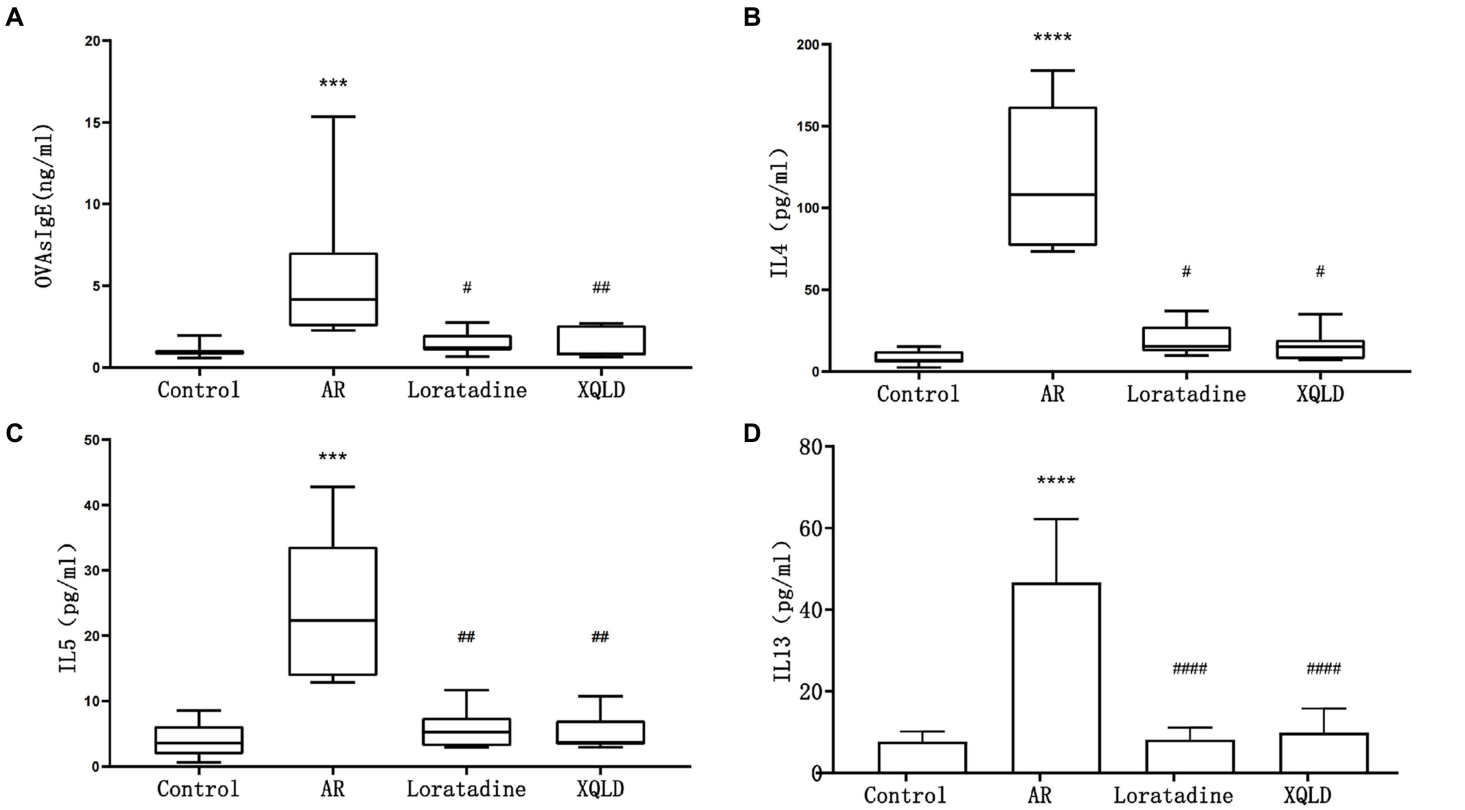
Serum ELISA results in mice - OVAsIgE **(A)**, IL4 **(B)**, IL5 **(C)**, and IL13 **(D)**. The significance analysis of the AR group is obtained by comparing it with the control group to obtain the *p*-value (^*^p < 0.05 and ^****^p < 0.0001). The significance analysis of the Loratadine and XQLD groups is obtained by comparing them with the AR group to obtain the *p*-value (^#^*p* < 0.05 and ^####^*p* < 0.0001). Control refers to the group where no drug treatment is administered after the modeling process.

On days 0, 7, and 14, mice in the blank control group received intraperitoneal injections of 300 μL phosphate-buffered saline (PBS). In contrast, the other three groups were intraperitoneally injected with 300 μL PBS containing 25 μg OVA and 1 mg aluminum hydroxide for sensitization. From days 22 to 28, mice in the blank control group underwent daily intranasal administration of 10 μL PBS per nostril. Conversely, the remaining three groups were locally sensitized through daily intranasal administration of a 50 mg/mL OVA solution (dissolved in PBS), also at a volume of 10 μL per nostril. On day 29, mice were humanely euthanized under anesthesia via cervical dislocation for subsequent sample collection. The establishment of the AR model can be affirmed by the presence of noticeable nasal symptoms in mice, including itching, sneezing, and nasal discharge (data shown in Figure 1), along with a significant elevation in serum levels of ovalbumin specific immunoglobulin E (OVAsIgE), IL4, IL5, and IL13 expression (data shown in Figure 2) (Liu et al., 2023).

**Figure 3:**
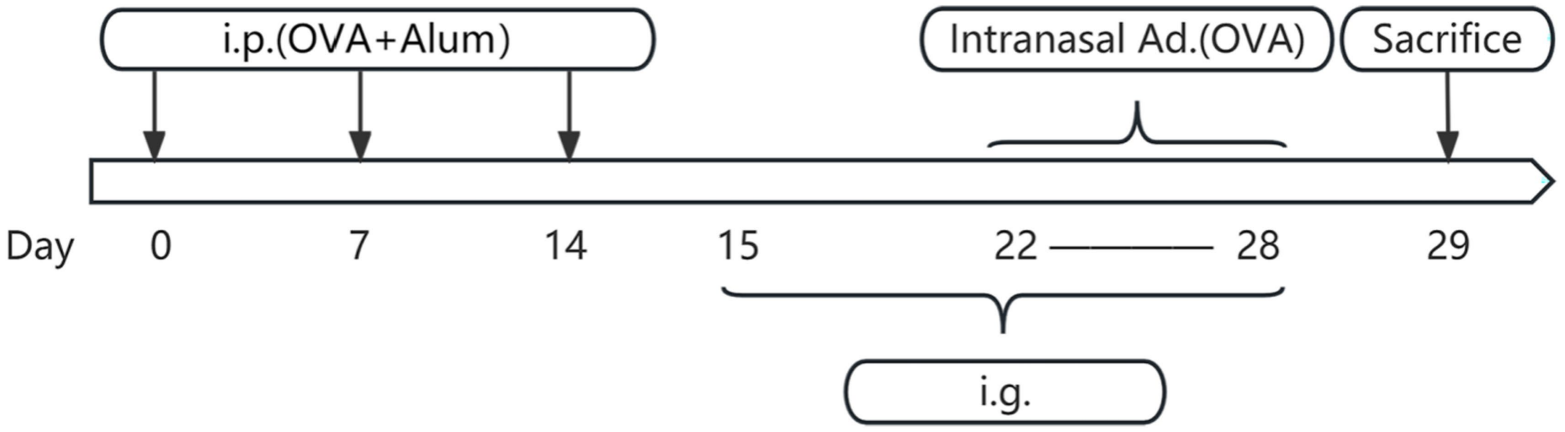
Flowchart of mouse model establishment and drug administration. Days 0–14 involve three basic sensitizations through intraperitoneal injection. Days 15–28 include daily drug intervention therapy on the stomach. Days 22–28 involve intranasal administration for nasal sensitization. Day 29 is the time for sampling. i.p., intraperitoneal injection; OVA, ovalbumin; Alum, aluminum hydroxide; Intranasal ad., intranasal administration; i.g., intragastric administration.

Concurrently, during the period from days 15 to 28: the blank control group received daily oral gavage of 100 μL PBS. The XQLD group received daily oral gavage of XQLD preparation at a dosage equivalent to the clinical standard (11.83 g/kg). The Loratadine group received daily oral gavage of loratadine at a dosage of 1.547 mg/kg (Figure 3).

### 2.2 XQLD preparation

Xiaoqinglong Decoction was administered to the mice at a dose equivalent to 1 times the clinical effective dose of XQLD, by oral gavage daily from day 15 to day 28. The specific dose was 11.83 g of XQLD crude herbs per kilogram of mouse body weight. The concentration of the XQLD liquid for gavage was 2.4 g/mL, with approximately 100 microliters administered to each mouse per day. The detailed information is as follows.

Table 1 presents comprehensive information on all the medicinal components contained within XQLD. The accuracy of plant names has been verified using data from http://www.theplantlist.org. These were purchased from the same batch at the First Affiliated Hospital of Guangzhou University of Chinese Medicine. XQLD was prepared using the water reflux method, a technique that has been previously utilized in our research. In these previous studies, we conducted an analysis of its drug components through Liquid Chromatography-Mass Spectrometry (LC–MS) analysis, thereby affirming the stability and reliability of the traditional Chinese medicine solution we prepared (Liu et al., 2023).

**Table 1:** Chinese medicinal herb contained in Xiaoqinglong decoction and the supplier information of all herbs.

Chinese naming of different ingredients	Full botanical plant names	Amount(g)	Manufacturer source	Batch number
Zhi Gan Cao	*Glycyrrhiza uralensis* Fisch.	9	The Chinese Medicine Material Processing Plant	G0419515
Ma Huang	*Ephedra sinica* Stapf	9	The Chinese Medicine Material Processing Plant	YPA900001
Bai Shao	*Paeonia lactiflora* Pall.	9	Guangdong Amagi Pieces of Chinese Medicine	190601
Wu Wei Zi	*Schisandra chinensis* (Turcz.) Baill.	12	Guangdong Amagi Pieces of Chinese Medicine	190301
Gan Jiang	*Zingiber officinale* Roscoe	9	Guangdong Amagi Pieces of Chinese Medicine	190901
Gui Zhi	*Neolitsea cassia* (L.) Kosterm.	9	Guangdong Amagi Pieces of Chinese Medicine	191001
Xi Xin	*Asarum heterotropoides* F.Schmidt	9	Kangmei Pharmaceutical Co., Ltd.	191200991
Fa Ban Xia	*Pinellia ternata* (Thunb.) Makino	12	Zisun Chinese Pharmaceutical Co., Ltd.	190504

### 2.3 Other drugs and devices

Loratadine tablets [Bayer Healthcare (Shanghai) Co., Ltd., National Drug Approval Number H10970410]. OVA (Sigma, United States, A5503-5G). Aluminum Hydroxide (Thermo Fisher Scientific, United States, 77161). Mouse OVAsIgE enzyme-linked immunosorbent assay (ELISA) Kit (Shanghai Jianglai Biotechnology Co., Ltd., JL20466). Mouse IL4 ELISA Kit (Shanghai Jianglai Biotechnology Co., Ltd., JL20266). Mouse IL5 ELISA Kit (Shanghai Jianglai Biotechnology Co., Ltd., JL20267). Mouse IL13 ELISA Kit (Shanghai Jianglai Biotechnology Co., Ltd., JL20247). DNA extraction kit (Omega Soil DNA Kit, D5625-01). High-fidelity DNA polymerase (NEB, M0491L). Gel recovery kit (Axygen, AP-GX-250). Fluorescence quantitative polymerase chain reaction (PCR) reagent kit (BioTek, United States, FLx800). High-fidelity DNA polymerase (NEB, M0491L). Gel recovery kit (Axygen, AP-GX-250). Fluorescence quantitative PCR reagent kit (BioTek, United States, FLx800).

Rotary evaporator (Shanghai Yarong Biochemical Instrument Factory, RE-2000A); Circulating water vacuum pump (Zhengzhou Changcheng Science and Technology Trade Co., Ltd., SHB-III); Fc-type ELISA reader (Thermo, United States); High-speed refrigerated centrifuge (Beckman Coulter, Inc., United States); PCR instrument (Bio-Rad, Germany, C1000).

### 2.4 Behavioral evaluation of mice

Throughout the duration of the experiment, mice were subjected to continuous monitoring encompassing various aspects of their general condition and behavior. This comprehensive evaluation included observations of their behavior, overall health, feeding and drinking patterns, defecation, and other general parameters. Specific attention was dedicated to the assessment of nasal symptoms, including nose rubbing, sneezing, and nasal secretions. Additionally, the frequency of sneezing and nose rubbing within a 10-min interval following the last stimulus was meticulously recorded to evaluate the drug’s influence on nasal symptoms.

### 2.5 Detection of serum OVAsIgE, IL-4, IL-5, and IL-13 in mice

Serum levels of mouse OVA-specific IgE (OVA-sIgE), IL-4, IL-5, and IL-13 were quantified employing commercially available ELISA kits specific to each analyte. Optical density measurements for each well were recorded at a wavelength of 450 nm. To determine the concentration of each sample, a standard concentration was plotted on the *x*-axis, and the corresponding optical density values were plotted on the *y*-axis in an Excel worksheet. Concentration values for each sample were calculated based on the curve equation derived from the standard curve.

### 2.6 Analysis of mouse gut microbiota

Mouse fecal samples were subjected to DNA extraction utilizing a DNA extraction kit. Following successful gel electrophoresis, PCR amplification was carried out employing a high-fidelity DNA polymerase. PCR products were subsequently collected and purified using a gel recovery kit, and sample concentrations were determined through fluorescent quantitative PCR to meet the requirements for high-throughput sequencing. The high-throughput sequencing process was outsourced to Novogene Co., Ltd., with a focus on the V3 + V4 regions and conducted as 2 × 300 bp paired-end sequencing on the MiSeq sequencing platform. The obtained sequencing results underwent rigorous quality control, assembly, and subsequent analysis of microbial diversity.

### 2.7 Statistical analysis

Data analysis and graphical representation were carried out using GraphPad Prism 8.0. For normally distributed continuous data, the results were expressed as X^−^ ± s, and bar graphs were utilized for visualization. In cases of non-normally distributed data, *M(P25 ~ P75)* represented the data, and box plots were employed for visualization. When continuous data adhered to normal distribution and homoscedasticity assumptions, intergroup comparisons were performed using one-way analysis of variance (ANOVA) followed by Dunnett’s test. In instances of non-normally distributed or heteroscedastic data, the Kruskal–Wallis test was applied, followed by Dunn’s test. Statistical significance was defined as *p* < 0.05. The correlation analysis was conducted using Genes Cloud Tools.^1^

## 3 Results

### 3.1 Mouse behavioral

The frequency of sneezing and nose rubbing episodes within 10 min following the last nasal administration was recorded for all groups of mice. Our findings revealed that the AR model group exhibited significantly more pronounced symptoms in comparison to the blank control group (*p* < 0.05), signifying evident nasal sensitivity symptoms in mice subsequent to OVA sensitization. However, in the treatment groups, mice displayed significantly reduced occurrences of sneezing and nose scratching when compared to the AR model group (*p* < 0.05). This observation suggests that both XQLD and Loratadine have the capacity to alleviate nasal sensitivity symptoms in AR-afflicted mice (Figure 1).

### 3.2 ELISA

In this investigation, we induced an allergic rhinitis (AR) mouse model through OVA sensitization, a process primarily characterized by IgE-mediated allergic inflammation driven by Th2 polarization. To confirm the successful establishment of the OVA-induced AR model and assess the therapeutic efficacy of the administered drugs, we employed ELISA assays to quantify the levels of serum OVAsIgE, IL4, IL5, and IL13 across all mouse groups. Our results demonstrated a significant elevation in serum OVAsIgE, IL4, IL5, and IL13 levels in the AR model group compared to the blank control group (*p* < 0.05), affirming the successful construction of the AR mouse model. Furthermore, following drug administration, all groups of mice exhibited a marked reduction in serum OVAsIgE, IL4, IL5, and IL13 levels (*p* < 0.05). These findings strongly suggest the efficacy of the drugs under investigation, namely XQLD and the positive control drug Loratadine, in the treatment of AR in mice (Figure 2).

### 3.3 Microbiota

#### 3.3.1 Sequencing depth and operational taxonomic units (OTUs)

**Figure 4:**
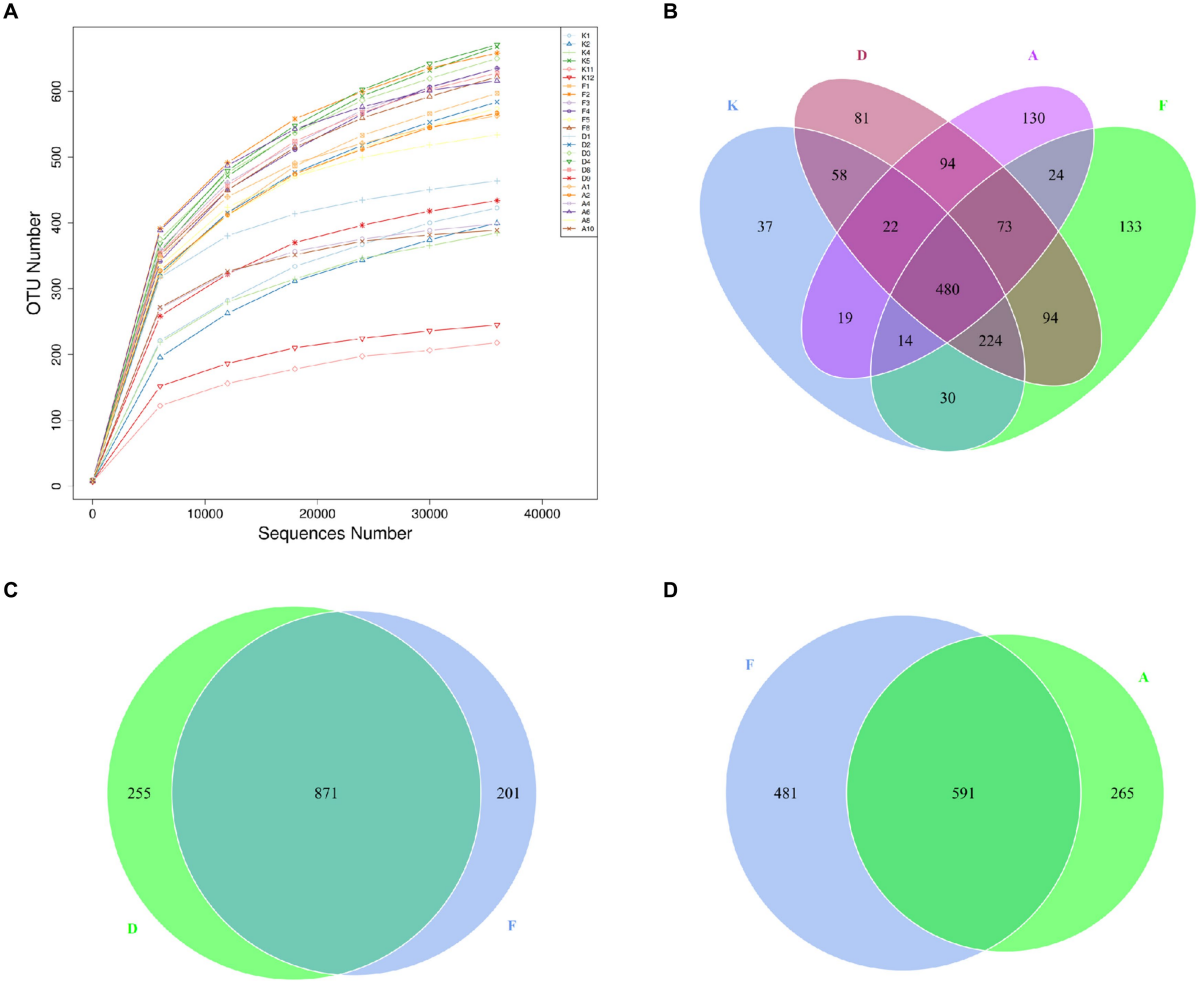
**(A)** Dilution curves of sequencing samples. *X*-axis: randomly extracted sequencing reads from samples, *Y*-axis: number of OTUs determined from the sequencing reads. **(B)** Venn diagram illustrating the distribution of OTUs among the four groups. **(C)** OTU Venn diagram comparing the AR model group with the Loratadine group. **(D)** OTU Venn diagram comparing the AR model group with the XQLD group (K, Control group; F, AR model group; D, Loratadine group; A, XQLD group).

In this investigation, fecal DNA was extracted from mice belonging to each respective group and subsequently subjected to high-throughput sequencing targeting the V3 + V4 region of bacterial DNA. The primary objective of this analysis was to assess the impact of XQLD on the gut microbiota composition of mice, particularly focusing on fecal microbiota diversity. Each OTU signifies a distinct bacterial species characterized by its unique 16S rRNA sequence. To ascertain whether the sequencing depth adhered to the required standards, dilution curves based on OTUs were constructed. Figure 4A illustrates these dilution curves for all samples under investigation. The outcomes derived from these curves reveal a consistent trend. As sequencing depth progressively increases, the curves for all samples gradually plateau. This phenomenon of plateauing curves signifies that further increases in sequencing depth would result in diminishing returns concerning the detection of new OTUs. Consequently, the sequencing results are deemed sufficient for accurately representing the existing sample diversity and align with the predefined analytical criteria.

A Venn diagram was utilized to assess the similarity and overlap of OTU numbers among various groups (Figure 4B). Figure 4C illustrates the OTU Venn diagram among the four groups. In total, 1,513 OTUs were identified across these groups, with 480 OTUs shared by all four groups, accounting for only 32% of the total OTUs. This finding indicates substantial differences in microbial community structures among these groups, with more than half of the bacteria being specific to particular groups. Figures 4C,D present the OTU Venn diagrams comparing the AR model group with the Loratadine group and the AR model group with the XQLD group, respectively. The results reveal that the Loratadine group has 255 unique OTUs, while the XQLD group has 265 unique OTUs. Despite similar quantities, these unique OTUs exhibit significant distinctions from those present in the AR model group.

#### 3.3.2 Alterations in the composition of gut microbiota in mice

**Figure 5:**
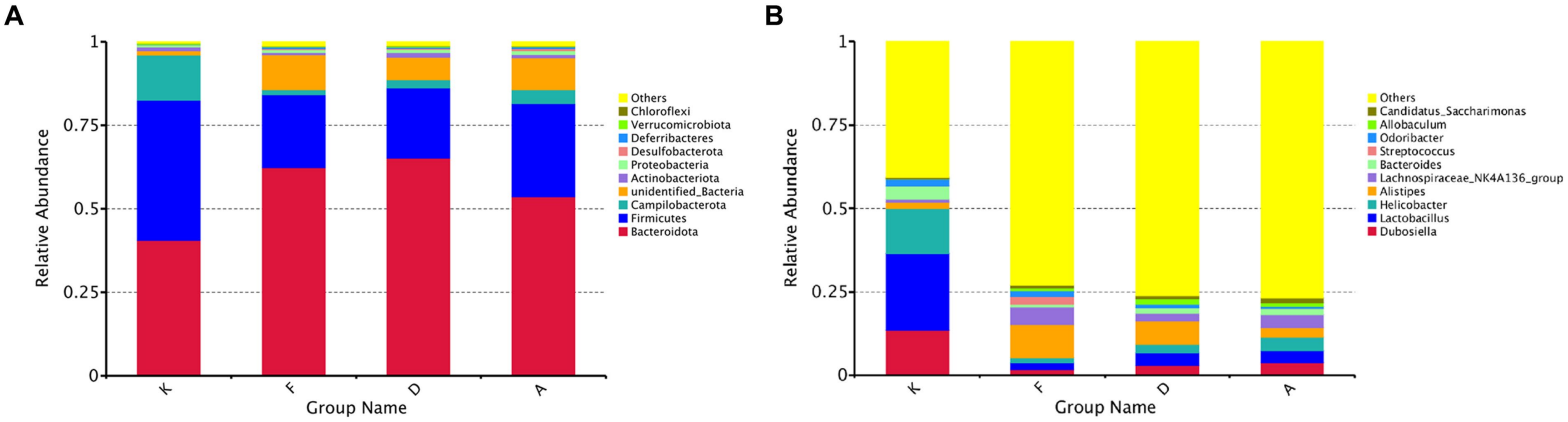
**(A)** Bar chart illustrating phylum-level relative abundances. **(B)** Bar chart illustrating genus-level relative abundances (K, Control group; F, AR model group; D, Loratadine group; A, XQLD group).

Through sequence alignment of measured OTUs with the National Center for Biotechnology Information (NCBI) database, we gained insights into the specific composition of microbial communities in each group. The results regarding community composition (Figure 5A) indicate that at the phylum level, dominant bacteria in the blank control group (K) primarily belong to *Firmicutes*, *Bacteroidota*, and *Campilobacterota*, constituting 96% of the total microbiota in this group. Following AR modeling, there is a substantial decrease in the proportion of *Firmicutes*, declining from 42 to 22%, a decrease in *Campilobacterota* from 13 to 1%, and an increase in *Bacteroidota* from 41 to 62%. Moreover, certain previously unidentified bacterial strains emerge, which were not present in the blank control group. This suggests that AR modeling disrupts the gut microbiota structure in mice to a certain extent. Subsequent intervention with Loratadine and XQLD induces further alterations in the microbiota. Particularly in the XQLD group, there is a resurgence in the abundance of *Firmicutes*, a reduction in *Bacteroidota*, and a trend toward restoring the community structure similar to that of the blank control group.

Figure 5B provides an in-depth analysis of microbial alterations at a more specific taxonomic level. In the fecal samples of the blank control group, the six most abundant genera were *Lactobacillus* (23%), *Helicobacter* (13%), *Dubosiella* (13%), *Bacteroides* (4%), and *Odoribacter* (2%), collectively representing 56% of the total microbiota. Following AR modeling, the overall proportion of the top 10 genera decreased from 59 to 27%, signifying a significant reduction in the abundance of the majority of dominant genera and highlighting the disruption of the original gut microbiota composition. After treatment with Loratadine and XQLD, further alterations in the microbiota composition were observed. The combined proportion of dominant genera, including *Lactobacillus*, *Helicobacter*, and *Dubosiella*, increased from 5% in the AR model group to 9% (Loratadine group) and 12% (XQLD group). Regarding the original dominant bacterial species in the gut, their abundance in the XQLD group was dramatically higher than in the loratadine group. However, these proportions remained notably different from those in the blank control group, which maintained a 50% proportion.

#### 3.3.3 Analysis of gut microbiota structural variations in mice

**Figure 6:**
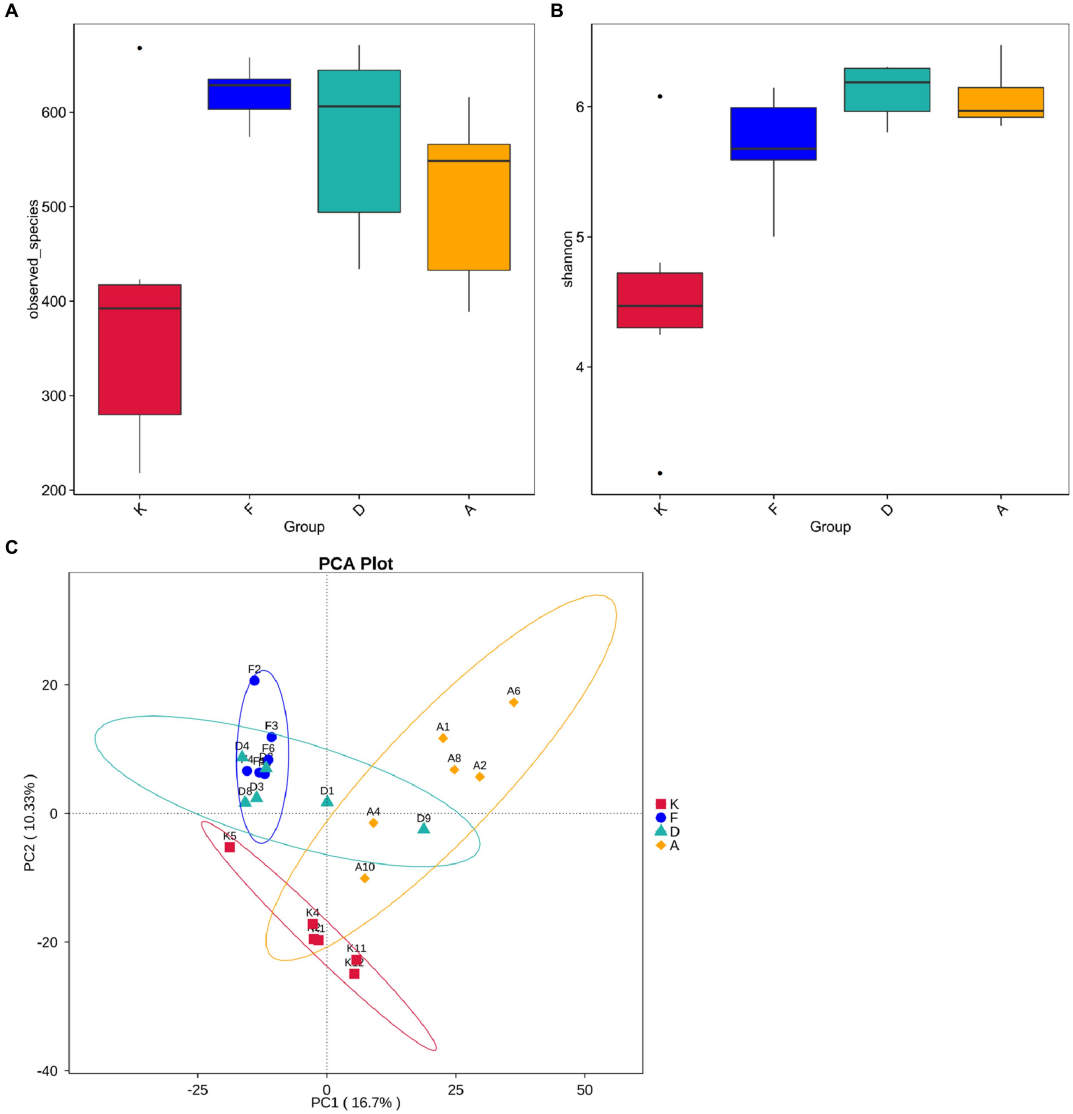
**(A)** Boxplot showing inter-group differences in observed species. **(B)** Boxplot demonstrating inter-group differences in Shannon index. **(C)** Beta diversity analysis of gut microbiota structure in mouse fecal samples (K, Control Group; F, AR Model Group; D, Loratadine Group; A, XQLD Group).

To further investigate the differences in community structure among the four sample sets, this study conducted α-diversity analyses. The Observed Species index reflects the species count present in each sample, while the Shannon index indicates species uniformity within the sample. As shown in Figure 6A, following AR modeling, the Observed Species index significantly increased from less than 400 in the blank control group to over 600, signifying a substantial rise in species richness. This difference between these two groups was highly significant (*p* < 0.01), possibly due to AR modeling altering the gut environment, leading to a decrease in the abundance of the original dominant microbiota and allowing external microbiota to colonize the gut more effectively. After the administration of XQLD, the Observed Species index showed a slight reduction, but t-tests revealed no significant difference compared to the blank control group. This indicates that, at the current dosage, XQLD’s impact on species abundance in the mouse gut may be limited. Figure 6B illustrates variations in the Shannon index among the groups. The AR Model, Loratadine, and XQLD groups all exhibited highly significant differences (*p* < 0.01) compared to the blank control group in terms of the Shannon index. However, when comparing the Loratadine and XQLD groups to the Model group, no significant differences were observed (*p* > 0.05), suggesting that drug administration had a minimal impact on microbiota evenness.

Beta diversity analysis is employed to evaluate diversity among habitats and can offer valuable insights into variations in community structure between different groups. Principal Component Analysis (PCA) results, as shown in Figure 6C, indicate a clear separation of data points between the AR model group and the blank control group, highlighting a significant distinction in community structure between these two groups. Despite loratadine intervention, there is still some overlap in data points with those of the AR model group, suggesting that the changes in community structure are not highly pronounced. In contrast, data points from the XQLD group are notably separated from the AR model group, indicating that the consumption of XQLD has a more pronounced impact on the gut microbiota compared to the use of loratadine.

#### 3.3.4 Prediction of functional changes in mouse gut microbiota

**Figure 7:**
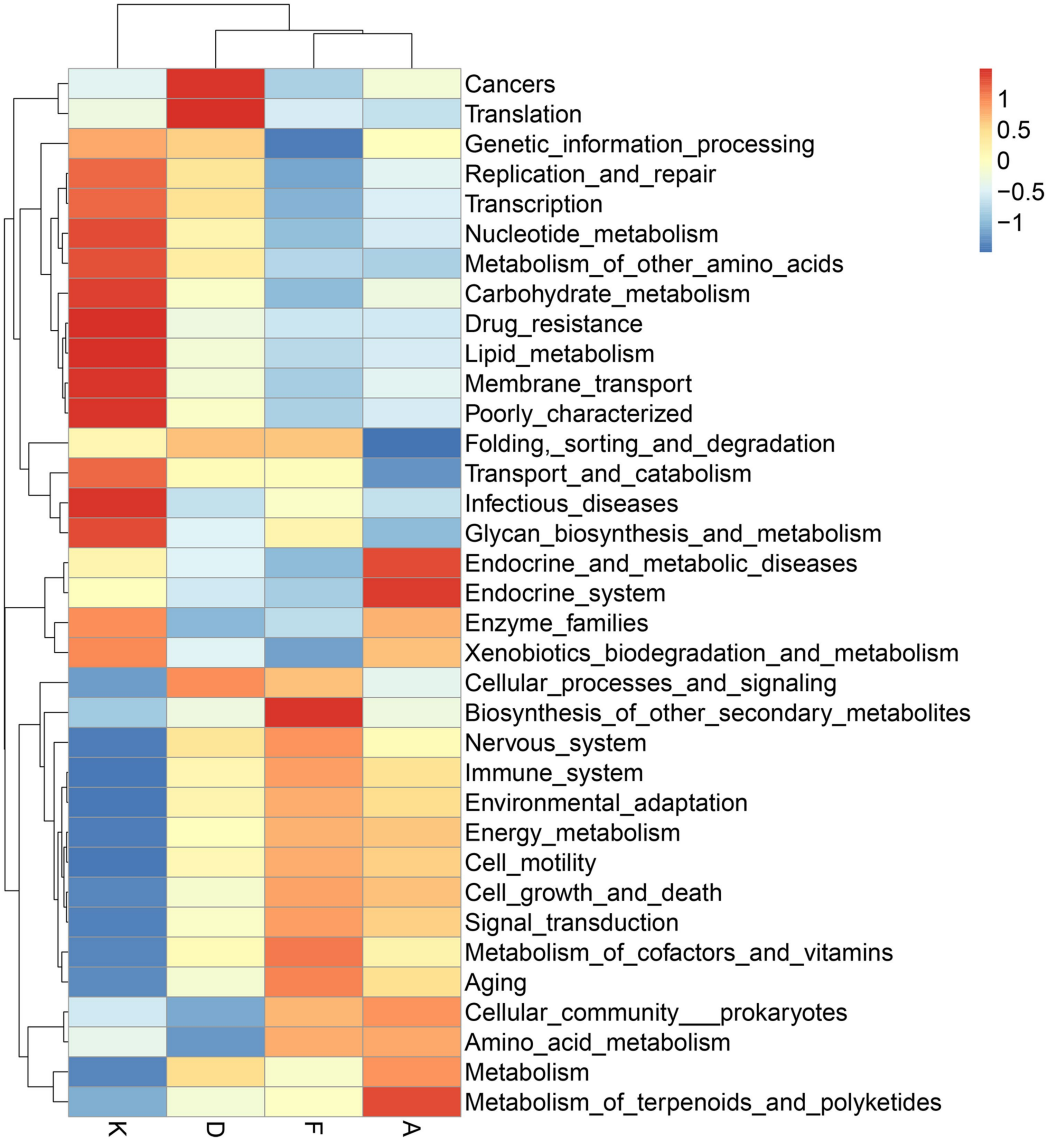
Functional predictions of gut microbiota utilizing the 16S Silva database (K, Control Group; F, AR model group; D, Loratadine group; A, XQLD group).

Following alterations in the gut microbiota, the physiological functions of its major microbial communities also undergo changes. While direct functional information about microorganisms cannot be obtained from 16S data, aligning the species information from each group with gene sequences in the KEGG database allows for an analysis of potential functions based on the whole-genome sequences of these species, facilitating the prediction of changes in gut microbiota functionality. As depicted in Figure 7, the modeling process led to shifts in various microbial functions. Notably, functions related to cancer and RNA transcription increased, while those linked to infectious diseases decreased. After intervention with XQLD and loratadine, the genes associated with cancer and RNA transcription returned to lower levels. However, genes associated with infectious diseases continued to decrease, possibly due to the drugs restricting the survival of pathogenic bacteria. Additionally, after drug administration, various functions related to cellular processes and metabolism displayed changes, highlighting the impact of microbiota alterations on gut microbiota functionality.

#### 3.3.5 The correlation analysis between the gut microbiota of mice and serum inflammatory factors

Correlation analysis was conducted between the gut microbiota (top 10 taxa at the phylum and genus levels across all samples) and serum inflammatory factors, providing insights into the correlation between changes in gut microbiota and variations in AR-related inflammation.

**Figure 8:**
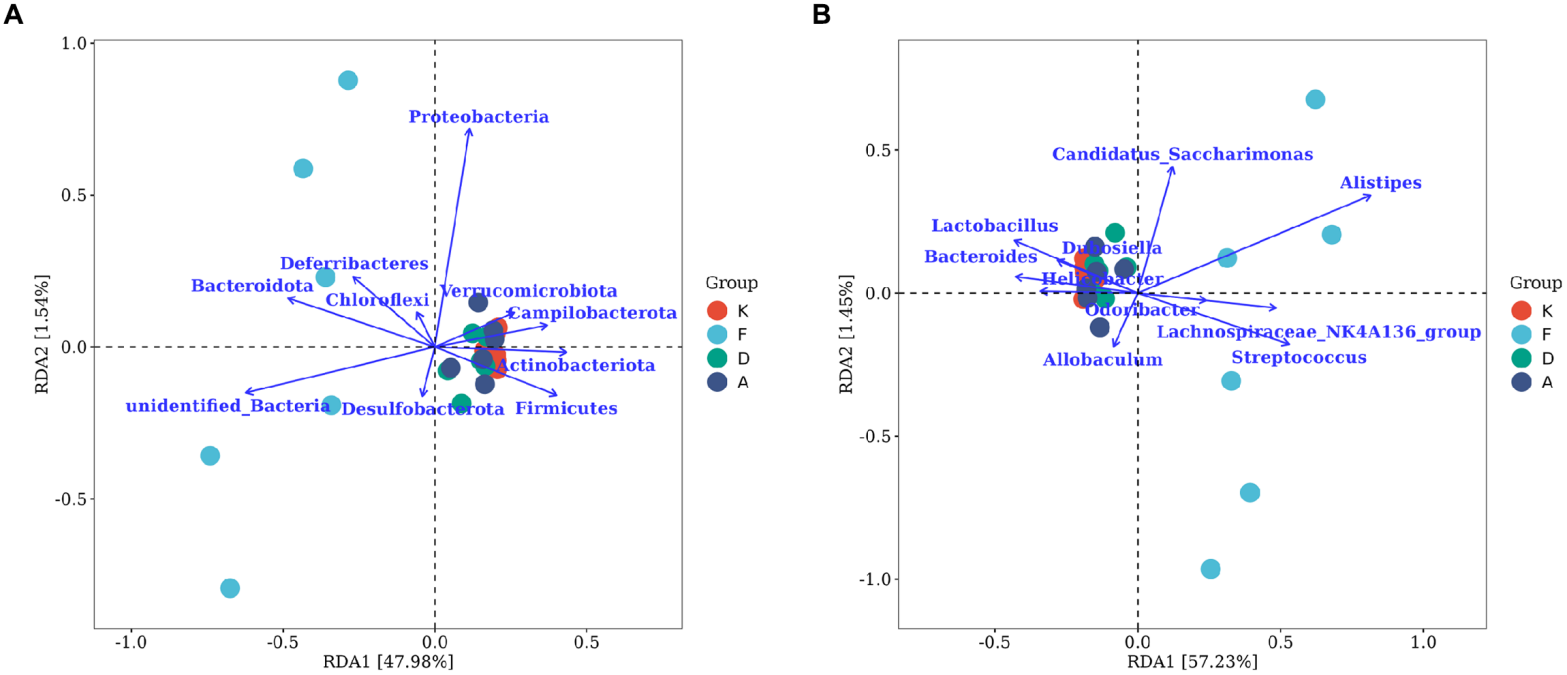
The RDA chart visualizes the correlation between gut microbiota and serum inflammatory factors in mice. **(A)** The top 10 taxa at the phylum level based on their relative abundance. **(B)** The top 10 taxa at the genus level based on their relative abundance. The plot displays serum inflammatory factors (points) and microbial taxa (blue arrows). Arrow length indicates microbial impact magnitude; longer arrows suggest greater influence. Arrow angles reflect taxon-axis correlation, smaller angles denote higher correlation. Sample projections on arrows approximate corresponding factor values. *p*-values above show significance of microbial changes, smaller *p*-values indicate more impact. Percentages next to axes represent variance explained (K, Control group; F, AR model group; D, Loratadine group; A, XQLD group).

Figure 8A represents the correlation analysis at the phylum lever. The arrows indicating *Actinobacteriota*, *Firmicutes*, and *Campilobacterota* at the phylum level align closely with the distribution of samples in the blank control group. Additionally, these arrows form smaller angles with the distributions of samples from the Loratadine group and XQLD group, suggesting a positive correlation. Conversely, a negative correlation is observed with the distribution of samples from the AR model group. Notably, *Bacteroidota* and *Deferribacteres* at the phylum level demonstrate a positive correlation with the AR model group, while displaying a negative correlation with the other groups.

Figure 8B represents the correlation analysis at the genus level. *Lactobacillus*, *Bacteroides*, and *Helicobacter* are the three microbial taxa most strongly correlated with the blank control group. They also display positive correlations with the Loratadine group and XQLD group, while exhibiting a negative correlation with the AR model group. On the contrary, *Allobaculum* and *Streptococcus* are genera that are most strongly correlated with the AR model group and negatively correlated with the other groups.

## 4 Discussion

The AR is typically characterized by symptoms such as sneezing and rhinorrhea, with its key pathological change being an IgE-mediated allergic inflammatory response induced by Th2 polarization (Lee et al., 2023). The onset and progression of AR involve numerous factors, with an imbalance in CD4+ T cell differentiation representing the central mechanism behind its allergic inflammatory response (Wise et al., 2023). Upon encountering abnormal antigenic stimulation, CD4+ T cells, specifically helper T cells (Ths), undergo aberrant differentiation. This leads to a bias toward Th2 cell development, subsequently resulting in the release of inflammatory cytokines such as IL4, IL5, IL9, and IL13. This alteration promotes IgE production, and the abnormal increase in IgE levels mediates the subsequent cascade of inflammatory reactions observed in AR. In this study, AR model mice not only exhibited pronounced nasal symptoms (*p* < 0.05) but also displayed a significant elevation in serum IgE, IL4, IL5, and IL13 expression (*p* < 0.05). XQLD effectively suppressed the upregulation of these cytokines and notably ameliorated sneezing and nose rubbing symptoms in the mice (*p* < 0.05). This molecular-level evidence underscores the therapeutic efficacy of XQLD in the treatment of AR.

In recent years, the role of epigenetic changes in the pathogenesis of AR has gained increasing recognition, with one of the primary epigenetic modifications driving AR development being the overexpression of histone deacetylases (HDACs) (Steelant et al., 2019). Our previous research has demonstrated that XQLD effectively regulates the skewed differentiation of Th2 cells and notably reduces the overexpression of HDAC1 and HDAC3 in AR mice (Liu et al., 2023). However, it’s crucial to emphasize that the primary active components in XQLD differ from recognized HDAC inhibitors. This suggests that XQLD’s inhibition of HDACs may occur through an indirect regulatory mechanism. Emerging evidence suggests that metabolites generated by gut microbiota, including lipids, amino acids, vitamins, short-chain fatty acids (SCFAs), lipopolysaccharides, and polysaccharide peptides, have the potential to induce changes in the host cell epigenome. Specifically, the production of SCFAs can effectively suppress HDAC activity, thereby modulating host cell processes. Our study’s community composition results (Figure 5A) reveal a significant reduction in the *Firmicutes* phylum within the gut of AR mice, coupled with an increase in the *Bacteroidetes* phylum. Members of the *Bacteroidetes* phylum are primary sources of acetic acid and propionic acid, whereas the *Firmicutes* phylum is recognized as the primary producer of butyric acid, a specific type of SCFA predominantly responsible for inhibiting the expression of HDACs (Xiao et al., 2022). These findings suggest that gut microbiota dysbiosis in AR mice may potentially disrupt SCFAs metabolism, favoring HDAC overexpression and thereby likely contributing to the initiation of inflammatory responses.

Following XQLD treatment, the gut microbiota in AR mice displayed a trend toward normalization. Specifically, at the phylum level, XQLD intervention resulted in an elevation of *Firmicutes* and a reduction in *Bacteroidetes*. This intervention contributed to a partial restoration of the community structure, aligning it more closely with that of normal mice, as depicted in Figure 5A. Our previous research (Liu et al., 2023) had demonstrated that XQLD effectively reduces the overexpression of HDACs 1, 3, and 4 in AR mice. Building upon the findings, it can be inferred that alterations in the microbiota may contribute to the observed downregulation of the aforementioned HDACs. Furthermore, following XQLD intervention, there is a noticeable trend toward the restoration of dominant genera such as *Lactobacillus*, *Helicobacter*, and *Dubosiella*, moving toward a composition similar to that of a normal microbiota. We also conducted a correlation analysis between gut microbiota and inflammatory factors, confirming that changes in microbial composition indeed have a certain impact on the development and recovery of AR-related inflammation. *Lactobacillus*, in particular, is a well-known gut probiotic naturally residing in the intestines of vertebrates and mammals. It possesses the ability to modulate the distribution of gut microbiota and counteract the colonization of pathogenic organisms (Tereza et al., 2023). *Helicobacter* and *Bacteroides* genera have also been previously documented as typical residents of the mouse intestinal tract (Vasapolli et al., 2019). The resurgence in the abundance of the mentioned dominant bacterial species is likely to benefit the restoration of immune regulatory functions within the gut-lung axis. This phenomenon may well be one of the factors contributing to the reduced frequency of post-treatment relapses observed with XQLD.

Additionally, this study conducted a comparative analysis with the commonly prescribed anti-allergic medication, loratadine. While loratadine is effective in mitigating AR symptoms, its long-term effectiveness is relatively suboptimal, often resulting in symptom recurrence upon discontinuation of medication (Nayak et al., 2017; Tenn et al., 2018). Examining the gut microbiota community structure at both the phylum and genus levels (Figure 5), the XQLD group exhibited a more pronounced tendency toward restoring the gut microbiota structure compared to the Loratadine group. Beta diversity analysis also reveals (Figure 6C) that after loratadine intervention, the sample points still overlapped with the AR model group, indicating less significant changes in the community structure. Conversely, the sample points in the XQLD group were distinctly separated from the AR model group, suggesting that the use of XQLD has a more profound impact on the gut microbiota, surpassing the effects of loratadine. This implies that these two drugs have distinct influences on the gut microbiota. Possibly, this is because XQLD has a more substantial impact on the gut-lung axis, contributing to its overall superior therapeutic effects. This could also be one of the reasons for the better long-term efficacy of XQLD. In summary, XQLD intake may induce changes in the gut microbiota, promoting the recovery of specific dominant bacterial species. However, whether this effect is beneficial for gut-lung axis and whether these alterations necessarily affect the progress of AR treatment remain topics worthy of further investigation.

## 5 Conclusion

This study conducted a investigation into the therapeutic effects of XQLD on AR mice, with a specific focus on elucidating the alterations in the gut microbiota following treatment. The results indicate that XQLD has a significant therapeutic impact on AR mice. It effectively suppresses the allergic inflammatory response, alleviates nasal symptoms, and restores normal nasal function. Moreover, during the AR modeling process in mice, there was a notable dysbiosis in their gut microbiota, characterized by a substantial reduction in the *Firmicutes* phylum and an increase in the *Bacteroidetes* phylum. However, after treatment with XQLD, this dysbiosis exhibited a trend toward recovery, resembling the microbiota composition in normal mice. XQLD likely exerts its influence on the gut-lung axis, thereby regulating gut microbiota dysbiosis and improving the allergic inflammatory response in AR. This could be one of the reasons for XQLD’s superior overall therapeutic effect and improved long-term efficacy. However, this proposed mechanism requires further validation through additional research.

In summary, this study elucidates the characteristic changes in the gut microbiota of mice influenced by XQLD, offering valuable insights for the clinical treatment and further development of XQLD.

## Data availability statement

The data presented in the study are deposited in the NCBI repository, accession number PRJNA1104586.

## Ethics statement

The animal study was approved by Ethics Committee of the Animal Experimental Center at the First Affiliated Hospital of Guangzhou University of Chinese Medicine. The study was conducted in accordance with the local legislation and institutional requirements.

## Author contributions

H-LL: Writing – original draft. H-FC: Writing – original draft. Y-DW: Visualization, Writing – original draft. Y-JY: Data curation, Writing – original draft. X-CH: Data curation, Writing – original draft. Z-ZL: Data curation, Writing – original draft. YR: Writing – review & editing. G-LW: Writing – review & editing.

## Footnotes

^1^ https://www.genescloud.cn
